# The role of *N*-glycosylation in kiwi allergy

**DOI:** 10.1002/fsn3.99

**Published:** 2014-03-19

**Authors:** María Garrido-Arandia, Amaya Murua-García, Aranzazu Palacin, Leticia Tordesillas, Cristina Gómez-Casado, Natalia Blanca-Lopez, Tania Ramos, Gabriela Canto, Carlos Blanco, Javier Cuesta-Herranz, Rosa Sánchez-Monge, Luis F Pacios, Araceli Díaz Perales

**Affiliations:** 1 Centre for Plant Biotechnology and Genomics U.P.M. – I.N.I.A., Campus de MontegancedoPozuelo de Alarcón, Madrid, Spain; 2 Allergy Service, Hospital Infanta LeonorMadrid, Spain; 3 Health Research Institute La Princesa (IP)Madrid, Spain; 4 IIS-Allergy Service, Fundación Jiménez DíazMadrid, Spain; 5 Biotechnology Department, ETSI de Montes, Technical UniversityMadrid, Spain

**Keywords:** Act d 2, allergen sensitization, carbohydrate complex determinant, *N*-glycosylation, plant food allergy, thaumatin-like protein

## Abstract

The physical, biochemical, and immunological characteristics of plant allergens have been widely studied, but no definite conclusion has been reached about what actually makes a protein an allergen. In this sense, *N*-glycosylation is an exclusive characteristic of plant allergens not present in mammals and it could be implied in allergenic sensitization. With this aim, we evaluated and compared the allergenic activity of the protein fraction and the *N*-glycan fraction of the thaumatin-like protein and the main kiwi allergen, Act d 2. The natural allergen, Act d 2, was deglycosylated by trifluoromethanesulfonic acid treatment; the *N*-glycan fraction was obtained by extended treatment with proteinase K. *N*-glycan- and protein- fractions were recognized by specific IgE of kiwi-allergic patients. By contrast, the sugar moiety showed a reduced capacity to activate basophils and T cells, but not dendritic cells derived from patients' monocytes. Related to this, the production of cytokines such as IL6 and IL10 was increased by the incubation of dendritic cells with sugar moiety. Thus, the sugar moiety plays a significant role in sensitization, inducing the activation of antigen-presenting cells, but it is the protein fraction that is responsible for the allergic reactions.

## Introduction

The prevalence of food allergies has increased in Western countries over the last few years. These are sometimes life-threatening and compromise the growth and development of children. In this population, many foods have to be avoided to prevent adverse reactions. The problem has come to be recognized as being more serious in recent decades because allergic sensitization, which is now known to be a strong predisposing factor for asthma, has become considerably more prevalent (Platts-Mills and Woodfolk 1997; Platts-Mills et al. 1997). Little is known about the causes of this trend. The hygiene hypothesis proposes that the increase in allergic diseases is due to excessive cleanliness during early stages of life. However, it does not explain why, at a molecular level, some foods, but not others, promote sensitization, and why some proteins are more allergenic than others (Lack 2010; Frei et al. 2011).

Much of the research on allergen immunogenicity has focused on structural properties of proteins, such as size, solubility, and stability, which all contribute to the activation of immune cells (Aalberse and Stapel 2001; Pomes and Chapman 2001). More recently, the focus has turned toward examining whether these proteins—or associated molecules—have innate immunostimulatory properties (Ghaemmaghami et al. 2001; Svensson et al. 2004). Allergens are encountered by the human immune system as part of complex mixtures of proteins, carbohydrates, and lipids. Our immune system has evolved mechanisms to recognize bacterial proteins in combination with pathogen-associated molecular patterns (PAMPs) that induce appropriate Th1 responses (Janeway and Medzhitov 2002; Herre et al. 2004). Thus, what makes a protein allergenic may be partly determined by the context in which it is found. For example, some molecular patterns have a Th2 adjuvant function. Among the well-studied Th2 PAMPs are the *N*-glycans (van Ree et al. 2000; Faveeuw et al. 2003; Shreffler et al. 2006; Chruszcz et al. 2010; Emara et al. 2010; Royer et al. 2010). *α*-Fucosylated and *β*-xylosylated complex mannose glycans are common in plant, insect, and worm, but not mammalian glycoproteins (Garcia-Casado et al. 1996; Kolarich and Altmann 2000; van Ree et al. 2000; Wilson et al. 2001). Therefore, these glycans are attractive candidates as nonself structures recognized by the innate immune system for inducing the Th2-skewed responses that contribute to clinical allergy in susceptible individuals.

In plants, the *N*-glycosylation of proteins begins with the transfer of the oligosaccharide precursor Glc_3_Man_9_GlcNAc_2_ into the endoplasmic reticulum. This precursor can subsequently be modified by glycosidases and glycosyltransferases during transport of the glycoprotein through the endoplasmic reticulum, the Golgi apparatus, and the vacuole. Depending on the accessibility of the glycan side chain, these enzymes can convert the precursor into high mannose-type *N*-glycans, and then into complex-type *N*-glycans with an *α* (1,3)-fucose and/or a *β*(1,2)-xylose residues. This *N*-complex glycan, highly enriched in mannose, has been associated with Th2 responses (Altmann 2007).

Members of the thaumatin-like protein (TLP) family are important plant allergens in several fruits, such as peach, apple, cherry, kiwi, olive, and banana, and in pollens such as cypress (Breiteneder 2004; Palacin et al. 2008, Palacin et al. 2011, Palacin et al. 2012). These proteins have molecular masses of 20–30 kDa, and a highly stable three-dimensional structure, due to their eight disulfide bridges. Many of them are glycoproteins and there has been much debate about the role of their *N*-glycan fraction in their allergic cross-reactivity (Palacin et al. 2008, Palacin et al. 2011, Palacin et al. 2012). Thus, in this study, TLPs were selected as a model to determine the role of *N*-glycan fraction in allergenic sensitization and its role in IgE cross-reactivity. To this end, we deglycosylated Act d 2, a common kiwi allergen (Palacin et al. 2008) from the TLP family, and purified its *N*-glycan fraction. The IgE reactivity of the protein and *N*-glycan fractions was studied by in vitro and ex vivo assay. The effect of Act d 2 deglycosylation on the activation of monocyte-derived dendritic cells (moDCs) was measured, as well as the upregulation of maturation and activation markers. The ability of the *N*-glycan fraction to induce T-cell proliferation was also determined.

## Materials and Methods

### Patients and sera

Sera were collected from 24 patients (25–55 years old; 58% female, 42% male) with allergy to kiwi fruit (Table 1). All patients had a well-documented clinical history of immediate allergic reactions after kiwi ingestion; a positive response in the skin prick test (SPT) to kiwi (ALK-Abello, Madrid, Spain), and to Act d 2 in glycerol:PBS (1:1; v/v) at 50 *μ*g/mL (Eaaci 1989).

**Table 1:** Clinical characteristics of 24 patients with kiwi IgE-mediated hypersensitivity

					Kiwi				
							
No.	Age	Sex	Atopy	Total IgE (kU/mL)	Symptoms	Prick–prick^1^	DBPCfC	Other food allergies	Pollen
1	33	f	EIA	39	EIA	5	nd	Mustar	Grasses
2	39	f	r, BA	12	U/AE	14	OAS/AE	Mustard peach, nuts, legumes	No
3	16	m	r	361	OAS	10	Negative	Mustard peach, nuts, banana	No
4	21	f	r	50	A	9	nd	Mustard, peach	No
5	31	f	r	49	A	7	nd	Peach, nuts	No
6	16	m	r, BA	221	U/AE	4	OAS/U	Mustard, peach, nuts	Grasses
7	34	m	r, BA	1516	OAS	10	OAS	Mustard, tomato	Grasses
8	27	m	r	322	U/AE	6	Negative	Peach, avocado, nuts	No
9	46	f	r	710	OAS	20	nd	Peach, nuts, corn	Grasses
10	33	m	r, BA	345	OAS	13	OAS	Mustard, peach	Grasses
11	45	f	r, BA	681	OAS	9	OAS	Mustard, nuts	Grasses
12	17	f	r, BA	130	OAS	9	nd	Peach	Grasses
13	16	m	r, BA	113	OAS	10	Negative	Mustard, nuts	No
14	31	f	r	36	A	7	nd	Mustard, peach, corn	Grasses, Betula
15	26	m	r	294	OAS	6	nd	Mustard	No
16	33	f	r	314	A	4	nd	Peach	Grasses
17	32	m	r	596	OAS	7	Negative	Mustard, nuts	No
18	36	m	EIA	23	OAS	12	nd	Rosaceae fruits, nuts	Grasses
19	20	f	r, BA	12	OAS	5	nd	Rosaceae fruits, banana, citric fruits, muskmelon, nuts	Grasses, Artemisia
20	12	f	r	456	OAS	16	nd	Rosaceae fruits, citrus fruits, nuts	Grasses, Platanus, Olea, Plantago
21	21	M	r	234	OAS	8	nd	Rosaceae fruits	Grasses
22	34	f	r	980	OAS	5	nd	Rosaceae fruits	Grasses
23	37	f	r, BA	nd	OAS	6	nd	Rosaceae fruits, nuts	Grasses
24	32	M	r	nd	OAS	9	nd	Rosaceae fruits, muskmelon, banana, mustard, nuts	Grasses, Artemisia

No, patient number; F, female; M, male; EIA, exercise-induced anaphylaxis; R, rhinitis; BA, bronchial asthma; U, urticaria; AE, angioedema; OAS, oral allergy syndrome; A, anaphylaxis; DBPCFC, double-blind placebo controlled food challenge; nd, not done.

1 Result of skin prick test expressed in mm of mean wheal diameter 15 min after puncture, as performed with fresh kiwi.

Written informed consent was obtained from all patients, and the ethics committees of the corresponding hospitals approved the study.

### Purification and characterization of the natural form of thaumatin-like protein Act d 2

Act d 2 was purified from *Actinidia deliciosa* fruits using the method published by our group (Palacin et al. 2008). N-terminal amino acid sequencing and matrix-assisted laser desorption/ionization (MALDI) mass spectrometry analysis were carried out using standard methods with an Applied Biosystems Procise 494A gas-phase sequencer (Foster City, CA) and a Biflex-III spectrometer with delayed extraction (Bruker-Franzen Analytik, Bremen, Germany). The purified allergen was quantified using a commercial bicinchoninic acid test (Pierce, Cheshire, UK).

For cell cultures, the absence of LPS in the samples was checked using anti-LPS antibodies (rabbit anti-*Escherichia coli* LPS; AbD Serotec, Kidlington, UK) and using THP1-XBlue cells (Invivogen, Toulouse, France). These cells are derived from the human monocytic THP-1 cell line and are transfected with a reporter plasmid expressing a secreted embryonic alkaline phosphatase (SEAP) gene under the control of a promoter inducible by the transcription factors NF-κB and AP-1.

### Deglycosylation by trifluoromethanesulfonic acid treatment (TMSF)

Deglycosylation of Act d 2 was carried out with TMSF following the protocol supplied with the PROzyme/Glyco Glycofree Chemical deglycosylation kit (Promyze, Hayward, CA), with minor modifications. The protein was solubilized in 50 *μ*L TMSF:toluene (5:20; v/v) and the reaction was carried out in an ethanol/dry ice bath, followed by incubation at −20°C for 4 h. TMSF was then removed with pyridine solution in a dry ice/ethanol bath for 15 min. The deglycosylated protein (dAct d 2) was neutralized by adding 0.5% ammonium bicarbonate and dialyzed (cutoff point, 10 kDa) against H_2_O and then freeze-dried. The deglycosylated protein was quantified using the bicinchoninic acid test. Mus a 4 (Palacin et al. 2010) (a significant banana allergen and nonglycosylated TLP) was used as the control (data not shown).

### Circular dichroism (CD)

Circular dichroism measurements were performed on a Jasco Model J-715 Spectropolarimeter (Japan Spectroscopic Co., Tokyo, Japan) using a 0.1-cm path length cell equilibrated at 20°C. Spectra were recorded for a wavelength range of 190–260 nm with 1-nm resolution at 50 nm/min scan speed and the average of three readings was calculated. The final spectra were baseline-corrected by subtracting the corresponding solvent spectra obtained under identical conditions. Results were expressed as the mean residue ellipticity (θ) for a given wavelength. The data were fitted using CDNN CD spectra deconvolution software (Applied Photophysics, Leatherhead, UK).

### *β*-1,3-Glucanase activity assay

For this assay, the method described by Menu-Bouaouiche et al. (2003) was followed using carboxymethylated-Pachyman (CM-Pachyman; Megazyme, Wicklow, Ireland) as substrate. Proteins (20 *μ*g) were incubated in 0.3 mL of 0.5% (w/v) CM-Pachyman in 50 mmol/L sodium acetate, 100 mmol/L NaCl containing 0.05% (w/v) sodium azide (pH 4.0 and 5.0). A quantity of 4 mL of 0.1% (w/v) tetrazolium blue, 50 mmol/L NaOH and 0.5 mol/L sodium potassium tartrate were added to 50 *μ*L of samples and then heated in boiling water for 3 min. The quantity of liberated d-glucose equivalents was measured as the absorbance at 660 nm. Assays were performed in duplicate. Activity was expressed as mkat/mg of protein (1 kat corresponds to the formation of 1 mol of d-glucose equivalent per second). The reaction curve was plotted to calculate the *V* values. *K*_m_ and *V*_max_ were determined from a standard Lineweaver–Burke plot.

### Inhibition assays of fungal growth

Antifungal activity was estimated as previously described. Spores of *Plectosphaerella cucumerina* (10^6^ spores/mL) were grown in 100 *μ*L of potato dextrose broth (Sigma, St. Louis, MO) in 96-well microplates for 48 h at 23°C. A quantity of 20 *μ*g/150 *μ*L of Act d 2 or dAct d 2 proteins was added to the culture medium. The medium with fungal suspension without added proteins was used as negative control. Antifungal activity was monitored as optical densities (OD) at 660 nm every 24 h.

### Isolation of the *N*-glycan fraction from Act d 2

Act d 2 was incubated with 5 *μ*g/mL of proteinase K (Invitrogen, Carlsbad, CA) for 24 h at 55°C and then concentrated with Amicon Ultra 3K (Millipore, Billerica, MA). The unretained volume containing the sugar moiety was dialyzed against H_2_O (cutoff point, 500 Da). The monomer composition of the *N*-glycan was confirmed by strong acid hydrolysis and mass spectrometry. The total glycan was identified, and the sugar content was quantified according to Dubois' method. The *N*-glycan fraction was measured by matrix-assisted laser desorption/ionization (MALDI-FTMS) mass spectrometry using standard methods in the Mass-Spectrometry Services from Complutense University (Madrid, Spain).

### Western-blot analysis

Samples (3 *μ*g of Act d 2 and dAct d 2) were separated by sodiumdodecyl sulfate polyacrylamide gel electrophoresis (SDS-PAGE) and replica gels were electrotransferred onto polyvinylidene difluoride (PVDF) membranes. After blocking (Sigma-Aldrich, St. Louis, MO), membranes were incubated with a pool of patient sera (1:2 dilution in PBS) overnight, washed three times with PBS-Tween 20 0.05% for 10 min, then incubated with goat antihuman IgE-peroxidase conjugate (Invitrogen; 1:5000 dilution in PBS). IgE-binding components were detected by means of chemiluminescence (Amersham Biosciences, Little Chalfont, UK). Alternatively, blocked membranes were immunodetected with rabbit polyclonal antibodies to peach TLP (1:10.000 dilution; 1 h; kindly provided by Dr. C. Pastor, FJD, Madrid, Spain), with anti-complex asparagine-linked glycan serum (Faye et al. 1993) (1:10.000 dilution; 1 h) or with anti-xylose and anti-fucose antibodies (also kindly provided by Dr. C. Pastor, FJD), and then treated with anti-rabbit IgG-phosphatase alkaline conjugate (Sigma; 1:5000 dilution) and developed with 5-bromo-4-chloro-3-indolyl phosphate/nitroblue tetrazolium (Sigma).

### Immunoassays

ELISA assays were carried out as previously described (Diaz-Perales et al. 2003) to determine specific IgE of 24 individual sera (1:2 dilution in PBS) from patients with kiwi allergy using kiwi extract (15 *μ*g/mL), purified Act d 2, deglycosylated Act d 2* or *N*-glycan fraction (5 *μ*g/mL). For assaying carbohydrate moiety, Corning's Carbo-BINDTM plates were used following the manufacturer's instructions (Costar, Corning, NY). Blocking solution (Sigma, Steinheim, Germany) without solid phase was used as a negative control. Bromelain was used as an allergenic sugar control. Those assays yielding ODs > 0.12 units (*n* = 9; mean blocking + 3 × SD =0.043 + 3 × 0.026 OD units) were considered positive. All tests were performed in triplicate.

### Basophil activation test (BAT)

This test was performed as previously described (Sanz et al. 2002a,b; Gamboa et al. 2007). After blood-cell separation, 50 *μ*L of each patient's cell suspension was incubated with 50 *μ*L of 20 *μ*g/mL of Act d 2, dAct d 2 or *N*-gly. In order to evaluate background basal values without stimulation (negative control), we added 50 *μ*L of stimulation buffer (20 mmol/L *N*-2 hydroxyethypeperazine-*N*-2-ethansulfonic acid [HEPES], 133 mmol/L NaCl, 5 mmol/L KCl, 7 mmol/L CaCl_2_, 3.5 mmol/L MgCl_2_, 1 mg/mL bovine serum albumin, pH 7.4), containing IL-3 (2 ng/mL) and heparin (5000 UI/mL; ROVI, Madrid, Spain) in the cell suspension. As a positive control, a monoclonal anti-IgE antibody was used at a final concentration of 1 *μ*g/mL. A positive response was considered for stimulation index (SI; antigen-specific response/basal level) ≥2.

### Peripheral blood mononuclear cell (PBMC) proliferation assays

Peripheral blood mononuclear cells were purified from six kiwi-allergic patients, as previously described (Tordesillas et al. 2009). They were freshly isolated from 50 mL of blood by Lymphoprep (Axis-Shield, Oslo, Norway) density gradient centrifugation. 2 × 10^5^ cells per well in 200 *μ*L were cultured in 96-well plates (Costar) for proliferation analysis in Roswell Park Memorial Institute (RPMI) medium (Invitrogen, Paisley, UK), supplemented with 10% (v/v) of fetal calf serum (Invitrogen), 0.02 mmol/L mercaptoethanol, 2 mmol/L of glutamine, and 10 mmol/L HEPES. To evaluate cell proliferation, PBMCs were labeled with 5,6-carboxyfluorescein diacetate succinimidyl ester (CFSE) (Lyons and Parish 1994; Parish et al. 1998) following the manufacturer's instructions (Invitrogen). PBMCs were grown in the presence of 20 *μ*g/mL of Act d 2, dAct d 2, *N*-gly or phytohemagglutinin-L from *Phaseolus vulgaris* (PHA; Roche, Mannheim, Germany). The results were considered positive when the SI—calculated as the ratio between %CFSE^low^ (with stimulus)/%CFSE^low^ (without stimulus)—was >2. Data presented are the values obtained corresponding to antigen concentration of 5 *μ*g/mL.

### Generation of antigen-presenting cells

Monocytes were purified from PBMCs of six kiwi-allergic patients and six healthy donors by positive selection using CD14 dynabeads (Dynal Biotech ASA, Invitrogen, Oslo, Norway), following the manufacturer's protocol. DCs were derived from monocytes by culturing the CD14^+^ fraction in complete medium RPMI (Invitrogen) with l-glutamine, 10% heat-inactivated fetal bovine serum (Lonza, Amboise, France), and 100 mg/mL antibiotics (streptomycin and penicillin; Invitrogen) following the manufacturer's instructions, and with 200 ng/mL rhIL4 and 100 ng/mL rhGM-CSF (Immunotools, Friesoythe, Germany), for 4–5 days at 5% CO_2_ and 37°C, as described previously (Gomez et al. 2012). Immature DCs (imDCs) derived from monocytes were incubated in complete medium at 5 × 10^5^ cells/mL in 24-well plates (Falcon BD Labware, Le Pont de Claix, France) with Act d 2, dAct d 2, and *N*-gly at 20 *μ*g/mL. LPS at 10 *μ*g/mL (Sigma) was used as a positive control. After 72 h of stimulation at 37°C in 5% CO_2_, treated and nonuntreated DCs were recovered and maturation was assessed by CD83 (Immunotech, Marseille, France), CD80 and CD86 upregulation (Immunotools), in an Accuri cytometer (BD Accuri Cytometers, Ann Arbor, MI). The maturation index (MI) was calculated as the ratio between stimulated and unstimulated DCs. A response was considered positive when MI > 2.

### Quantification of cytokine expression

The expression of the cytokines IL-6, IL-10, TNF*α*, and IL-1*β* of moDCs was determined by real-time PCR, after incubating the cells with 20 *μ*g Act d 2, dAct d 2, and *N*-gly for 72 h. Cells were recovered and mRNA was isolated according to the Qiagen- RNeasy protocol (Qiagen, Valencia, CA) and stored at −80°C. RT-PCR was performed as previously described (Lopez-Torrejon et al. 2005; Tordesillas et al. 2011). cDNA was amplified using the Power SYBR Green PCR Master Mix (Applied Biosystems) according to the manufacturer's recommendations and run on an Applied Biosystems 7300 real-time detection system (Applied Biosystems), using previously described primers (Brown and McIntyre, 2011; Oberbach et al. 2011; Meiler et al. 2008; Soyka et al. 2012). Real-time PCR conditions were as follows: 10 min at 95°C, 15 sec at 95°C and 45 sec at 60°C (40 cycles). The level of IL-6, IL-10, TNF*α*, and IL-1*β* mRNA expression was normalized with endogenous control EF-1 (44) and relative quantification was performed using the comparative threshold cycle method (2^−DD*C*t^), as described by Livak and Schmittgen (2001). The changes in gene expression were calculated with respect to the untreated cells. All amplifications were carried out in triplicate, and results were derived from four different assays.

### Statistical analysis

Data were expressed as means ± SDs of two or more independent experiments. Statistical analysis was performed using SPSS 17.0 (IBM España, Madrid, Spain) and Statgraphics Centurion XVI (Madrid, Spain). Comparison between natural Act d 2 and protein/sugar fractions was carried out using Wilcoxon paired samples test or Mann–Whitney *U* test. A level of significance lower than 5% (*P* < 0.05) was considered to be significant in all analyses.

### Molecular modeling

The structural model of Act d 2 protein was constructed by homology modeling with SwissModel (Arnold et al. 2006) using the crystal structure of banana fruit thaumatin (PDB code 1Z3Q, sequence identity 77.11%) as the template. The structure of the sugar identified by mass spectrometry was constructed with Sweet2 using the corresponding symbol input generated with the DB Glycosuite database. The geometry of the final protein–*N*-glycan complex was assembled and refined with Chimera (Pettersen et al. 2004).

## Results

### The enzymatic activity of Act d 2 was not diminished by treatment with TMSF

**Figure 1:**
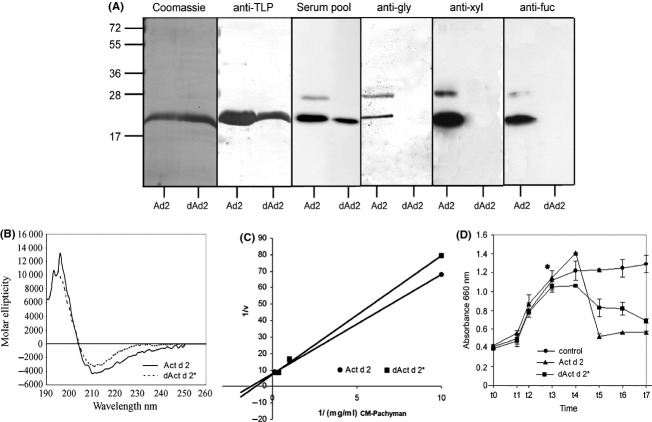
Comparison of Act d 2 and dAct d 2 (TMSF-treated Act d 2). (A) Coomassie-stained immunodetection with serum pool from kiwi-allergic patients (serum pool), or with rabbit polyclonal antibodies against thaumatin-like proteins (anti-TLP), plant complex glycans (anti-*N*-gly), xylose or fucose (anti-xyl and anti-fuc). Act d 2 (Ad2) and dAct d 2 (dAd2). (B) Secondary structure composition of Act d 2 and dAct d 2, calculated from their far-ultraviolet circular dichroism data. (C) *β*-Glucanase activity as a function of carboxymethylated-Pachyman concentration in a Lineweaver–Burke plot. (D) Antifungal activity. Spores from *Plectospharella cucumerina* were grown under standard conditions. Time corresponds to days after inoculation. Growth was measured as absorbance at 660 nm.

To determine the role of the *N*-glycan fraction in the allergenic activity of Act d 2, the natural protein purified from kiwi was deglycosylated (dAct d 2) by means of TMSF (Fig. 1). When subjected to SDS-PAGE, we found that both forms—Act d 2 and dAct d 2—migrated as a band with a similar molecular mass (Fig. 1A). The IgE-binding capacities of both forms were compared by Western blot using a pool of sera from kiwi-allergic patients (Fig. 1A, serum pool). In addition, epitopes recognized by anti-TLP antibodies were also retained after treatment (Fig. 1A, anti-TLP). However, no recognition was detected in dAct d 2 by antibodies produced against *N*-plant complex glycans (anti-*N*-gly), antibodies against xylose (anti-xyl) or by antibodies against fucose (anti-fuc), suggesting that deglycosylation treatment successfully removed the glycan fractions (Fig. 1A, anti-Ngly, anti-xyl, and anti-fuc).

In the same way, the far-ultraviolet CD (far-UV CD) of deglycosylated protein, dAct d 2, showed an overlapping spectrum with no differences compared to the natural protein, Act d 2 (Fig. 1B; Mann–Whitney *U* test, *P* = 0.312).

The enzymatic activity of the deglycosylated protein was measured by the *β*-glucanase assay using CM-Pachyman as substrate (Fig. 1C). dAct d 2 and Act d 2 exhibited similar activities, with no significant differences (Mann–Whitney *U* test, *P* = 0.121). The natural and deglycosylated forms had overlapping kinetic curves (*K*_m_ [Act d 2] = 10.8 mg/mL; *K*_m_ [dAct d 2*] = 10.81 mg/mL). The deglycosylated form preserved the ability to inhibit the growth of *P. cucumerina*, although this inhibitory capacity was lower than in the case of the natural protein (34.3% vs. 51.0% for dAct d 2 and Act d 2, respectively (Fig. 1D). These differences were not statistically significant (Mann–Whitney *U* test, *P* = 0.403).

### The *N*-glycan fraction of Act d 2 comprised a mixture of sugars

**Figure 2:**
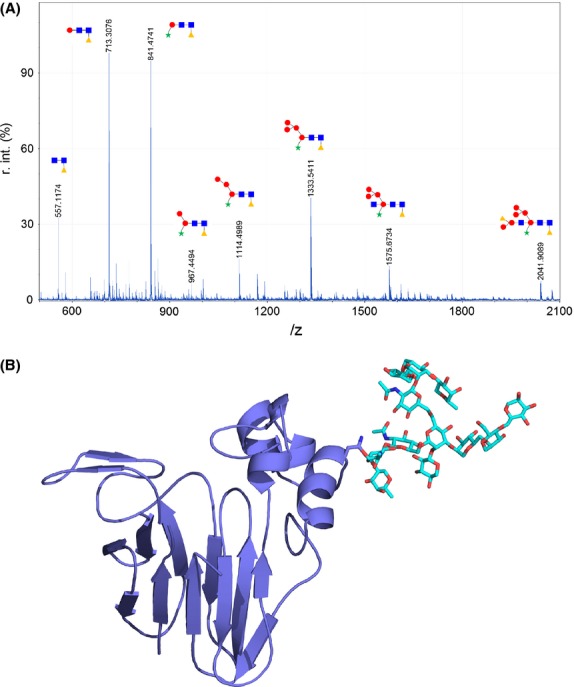
(A) *N*-glycan structures detected by mass spectrometry. Circles correspond to mannose, squares to *N*-acetylglucosamine, triangles to fucose, and stars to xylose. (B) Ribbon diagrams of Act d 2 showing the main sugar moiety as stick-like structures.

To study the contribution of *N*-glycan to IgE recognition of Act d 2, this fraction was isolated by extensive treatment of the natural allergen with proteinase K. The purity of the sugar fraction was confirmed by thin layer chromatography (data not shown) and mass spectrometry (Fig. 2A), in which no protein contamination was detected. The monomer composition of the *N*-glycan was analyzed by extensive acidic treatment and mass spectrometry. The sugar motif of Ac t d 2 consisted of monomers of mannose, *N*-acetyl glucosamine, xylose, and fucose, suggesting the presence of a typical *N*-plant glycosylation.

On the basis of molecular weights obtained by mass spectrometry, seven putative forms of *N*-glycan fractions were proposed by using the public DB Glycosuite database after selecting the 2041.9089 peak. The 3D structure of this selection is the sugar moiety linked to the structural model of Act d 2 protein represented in Figure 2B.

### The IgE-binding capacity of Act d 2 depended on both *N*-glycan and protein fractions

**Figure 3:**
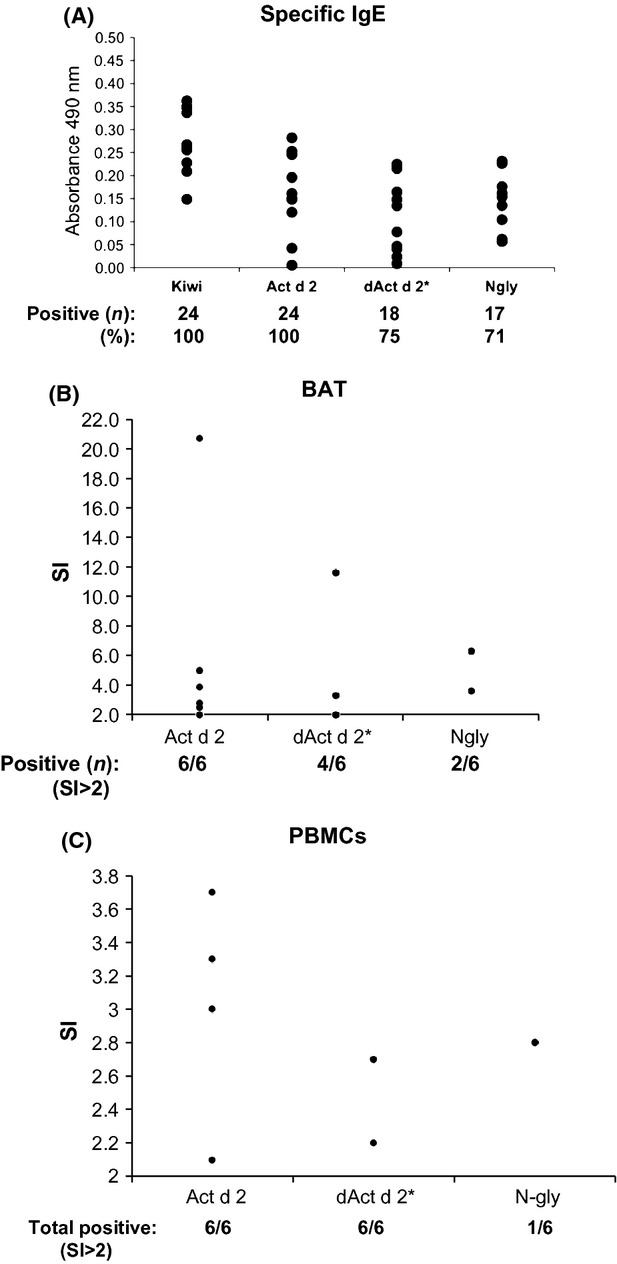
(A) Specific IgE levels in optical density units of kiwi extract, Act d 2, dAct d 2 and *N*-glycan fraction. Individual sera from 24 kiwi-sensitized patients were used. (B) Basophil activation tests in kiwi-allergic patients. The stimulation index (SI) is shown, calculated as% treated basophils/% untreated basophils. Responses involving 15% activated basophils and SI 2 were considered positive. (C) Activation of peripheral blood mononuclear cells (PBMCs). Positive SI responses (SI 2) of PBMCs from kiwi-allergic patients to Act d 2, dAct d 2, and *N*-gly is represented.

Act d 2, dAct d 2, and the *N*-glycan fraction (*N*-gly) were used as a solid phase to determine the specific IgE levels of single sera from 24 kiwi-allergic patients. Positive response to naïve allergen (Act d 2) was observed in all patients, while 16 of them (66.7%) recognized both fractions separately (Fig. 3A). Bromelain (Ana c 2)—a glycoprotein used as CCD control—gave a positive response in only four (17%) of these patients (data not shown).

Ex vivo, Act d 2 was able to activate basophils from kiwi-allergic patients, as well as the protein fraction (dAct d 2). By contrast, *N*-glycan fraction showed a strong reduction in this capacity—it was able to activate basophils of only two out of the six patients (Fig. 3B).

### *N*-glycan fraction showed a reduced capacity to activate kiwi patients' PBMCs, but could induce the maturation of monocyte-derived dendritic cells

**Figure 4:**
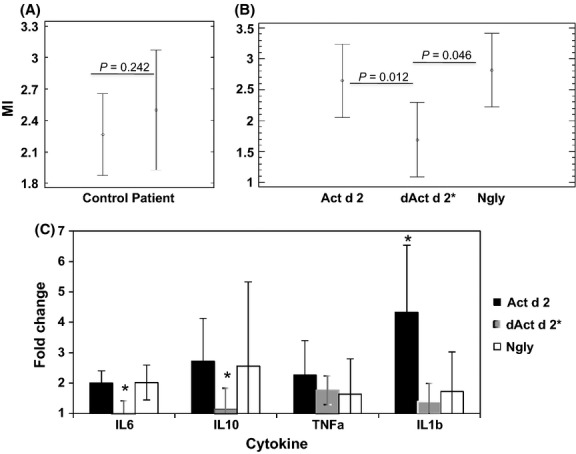
Phenotype of monocyte-derived dendritic cells (DCs). (A, B) Maturation index (MI, average) of monocyte-derived DCs from healthy donors (Control) and kiwi-allergic patients (Patient), after stimulation with natural protein (Act d 2; 20 *μ*g/mL), protein fraction (dActd2; 20 *μ*g/mL), and *N*-glycan fraction (*N*-gly; 20 *μ*g/mL). The means of five experiments are shown. Comparison between Controls and Patients (A). Comparison among different fractions (B). Data were analyzed by the Wilcoxon test or Mann–Whitney *U* test, respectively. (C) Cytokine profile. Changes in the production of Il-6, IL-10, TNF*α*, and IL-1*β* were measured by quantitative PCR. Amplifications were performed in triplicate and four independent assays. Means and SD (bars) are shown. Data were analyzed by Wilcoxon (with values of *P* < 0.05 considered significant).

Peripheral blood mononuclear cells from six kiwi-allergic patients were cultured either in the presence of the natural protein (Act d 2) or together with one of the isolated fractions, that is, the protein (dAct d 2*) or the *N*-glycan (*N*-gly) (Fig. 4). The protein fractions were able to activate lymphocytes in the same way as the natural protein did. Nevertheless, the capacity of *N*-glycan fraction was considerably reduced. Curiously, only PBMCs from one out of the six patients were capable of activating T-cell proliferation in the presence of sugar.

Monocyte-derived dendritic cells from six healthy donors and six allergic subjects were grown in the presence of increasing concentrations of deglycosylated protein (dAct d 2), *N*-glycan fraction (*N-*gly) and the natural allergen (Act d 2) (Fig. 4A). When the response of moDCs from controls and patients were compared, there were no statistically significant differences (Mann–Whitney *U* test, *P* = 0.2425).

Both sugar and naïve allergen induced the expression of the maturation markers CD80, CD83 and CD86, with no significant differences observed among them (Mann–Whitney *U* test, *P* = 0.2080). By contrast, the protein fraction dAct d 2 showed a reduced capacity to induce maturation of moDCs, showing statistical differences with respect to the natural allergen (Mann–Whitney *U* test, *P* = 0.0120) and *N*-glycan fraction (Mann–Whitney *U* test, *P* = 0.0460).

Finally, the cytokine expression levels of moDCs from patients were quantified. A statistically significant increase in the level of IL-6, IL-10, TNF*α*, and IL-1*β* was observed in treated moDCs compared to untreated moDCs (Wilcoxon test, *P* > 0.05; Fig. 4c).

In addition, the natural allergen and the sugar fraction induced higher levels of expression of IL6 and IL10, showing significant differences compared to the deglycoxylated protein. By contrast, the natural allergen was more active inducing the production of TNF*α* and IL1*β*.

## Discussion

Plant allergens are proteins belonging to a limit group of families, but the reason for their allergenicity remains unknown. The immunoactive regions of many of these proteins (B- and T-cell epitopes) have been studied, as have their heat and protease resistance properties. However, the day we can predict whether a protein is allergenic remains a long way off.

In order to understand the sensitization mechanism, in this study we evaluated the contribution of *N*-glycosylation in allergenic activity, taking Act d 2 (a TLP and a clinically significant allergen of kiwi) as a model. We isolated the protein and *N*-glycan fractions of this allergen. The deglycosylation treatment of Act d 2 did not alter its enzymatic activity or its antifungal capacity, and the far-UV CD indicated that its secondary structure was maintained.

The characterization of the *N*-glycan fraction of Act d 2 revealed a mixture of sugars. Mass spectrometry showed at least seven peaks between 1100 and 3000 Da. Searches of specialized sugar databases suggested several possible structures. The heterogeneity of these types of sugar could interfered in the match of TLPs as unique bands by SDS-PAGE, which may often be misinterpreted as a lack of purity in the samples (kiwi, peach) (Palacin et al. 2008, 2011, 2012).

Generally, plant glycoproteins prepared from biological sources are characterized by a mixture of trimmed and untrimmed *N*-glycans (van Ree et al. 2000). Earlier reports showed that xylose is not always equally well bound to specific antibodies (Altmann 2007), and suggested that the presence of *α*-(1,3)-mannose on M3X(F) structures might physically interfere with the binding. When *N*-glycan structures of the three major aeroallergens, Ole e 1, Ara h 1, and Lol p 11 were analyzed it was found that each one corresponded to at least two isoforms of sugar. The most extreme case was that of Ara h 1, which was found to be associated with at least four types of glycosylation, varying in their mannose and xylose content (van Ree et al. 2000). The same study demonstrated the pivotal role of the substitution of *α*-(1,3)-fucose and *β*-(1,2)-xylose in the IgE-binding capacity of the complex plant *N*-glycans. In our case, the monomer composition of the *N*-glycan fraction was confirmed by strong acid hydrolysis and mass spectrometry. This fraction contained monomers of mannose, *N*-acetyl glucosamine, xylose, and fucose, suggesting the presence of typical *N*-plant glycosylation.

The analysis of the IgE-binding capacity of the protein and *N*-glycan fractions revealed that most patients recognized both fractions. More than 77% of kiwi-allergic patients presented specific antibodies against both fractions, as shown by ELISA assays. By contrast, this recognition was not based on *N*-glycosylation cross-reactivity, because the bromelain (Ana c 2), commonly used as a glycoprotein control, was recognized by fewer than 17% of patients. In other words, *N*-glycan fraction was specifically IgE recognized.

However, this specific IgE to *N*-glycan fraction was shown not to play an important role in the basophil activation of patients to kiwi. This suggests that many patients may be sensitized to *N*-complex sugars of plants, but suffer no symptoms when they are exposed to them. CCDs are clinically irrelevant for most of the patients. According to the data, in a minor, but significant fraction of patients (10%) CCDs sensitivity might play a role in clinical allergy.

Conversely, we observed that the *N*-glycan fraction could induce maturation of antigen-presenting cells, such as moDCs, but could not activate PBMC proliferation. In fact, the culture of *N*-glycan fraction with moDCs induced the production of cytokines such as IL6 and IL10. These results suggest that the sugar may be able to stimulate an immune response from antigen-presenting cells, but the protein fractions (i.e., the natural or deglycosylated protein) could only induce a T-cell response.

The mechanism by which the *N*-glycan fraction can be recognized by dendritic cells is well understood (Altmann 2007). Many of the C-type lectin-like receptors that are found in DCs function mainly as antigen uptake receptors, including mannose receptor (MR, CD206), DCSIGN (CD209), and DEC-205 (CD205). In the case of Fel d 1—the principal allergen of cat dander—the MR plays a key role in the uptake by antigen-presenting cells (Emara et al. 2010), whereas Ara h 1 is recognized by dendritic cell-specific ICAM grabbing non-integrin (DC-SIGN), inducing a Th2 response (Shreffler et al. 2006). The different receptors involved in each case could be due to the sugar composition of the allergens. Fel d 1 presented mainly a mannose complex glycans (Emara et al. 2010) compared with Ara h 1, which had a typical *N*-glycan structure that was rich in fucose and xylose. It is possible that the mechanism involved in recognizing the *N*-glycan fraction of Act d 2 is comparable to that of Ara h 1.

In summary, this study shows the importance of the *N*-glycan fraction in the allergic sensitization to Act d 2. *N*-glycan does not appear to contribute to T-cell activation or the allergic cellular response. In summary, *N*-complex plant glycosylation may play an essential role in the activation of antigen-presenting cells and appears to trigger a proinflammatory response contributing toward the development of a Th2 response. By contrast, once the allergy is established, the *N*-glycan involvement in the induction of symptoms as well as in T-cell activation would be negligible. These findings provide useful information that is likely to influence current allergy immunotherapy strategies.
